# Phase angle at bioelectric impedance analysis is associated with detrimental sperm quality in idiopathic male infertility: a preliminary clinical study

**DOI:** 10.3389/fendo.2024.1354733

**Published:** 2024-04-24

**Authors:** Annalisa Liprino, Filippo Giacone, Debora Lombardo, Maria Giovanna Asmundo, Giorgio Ivan Russo, Ali Saber Abdelhameed, Sebastiano Cimino, Antonino Guglielmino, Sandrine Chamayou

**Affiliations:** ^1^ Unità di Medicina della Riproduzione, Centro HERA, Catania, Italy; ^2^ Urology Section, Department of Surgery, University of Catania, Catania, Italy; ^3^ Department of Pharmaceutical Chemistry, College of Pharmacy, King Saud University, Riyadh, Saudi Arabia

**Keywords:** bioelectric impedance analysis, male infertility, phase angle, semen analysis, sperm DNA fragmentation

## Abstract

**Background:**

In 2020, 38% of adults were affected by obesity, while infertility globally affected 1 in 6 people at some stage of their lives.

Body mass index (BMI) provides an easy but occasionally inaccurate estimation of body composition. To achieve a more precise assessment, bioelectric impedance analysis serves as a validated tool that administers electrical energy through surface electrodes. Phase angle as a function of the relationship between tissues resistance and reactance, is a trustworthy predictor of body composition and cell membrane integrity.

**Objectives:**

We aim to assess whether there is an association between phase angle and seminal parameters, as well as sperm DNA fragmentation percentage.

**Design:**

Semen samples of 520 idiopathic infertile patients were analyzed according to 2021 World Health Organization guidelines and evaluated for sperm DNA fragmentation rate. Each participants underwent bioelectric impedance analysis.

**Results:**

Median age was 40 years old, median BMI was 26.3 kg/m2, median phase angle was 6.2°. In the logistic regression analysis adjusted for age and total intracorporeal water, phase angle (continuous) was significantly associated with oligozoospermia (odds ratio [OR]:0.4; p<0.01) and sperm morphology (OR: 0.65; p=0.05) and slightly with sperm DNA fragmentation (OR: 0.98; p=0.07). In subgroup analysis, the logistic regression analysis adjusted for the mentioned parameters showed that a phase angle between 6.2 and 7 (°) (OR: 0.63; p=0.02) and >7 (°) (OR: 0.12; p<0.01) were associated with a reduced risk of oligozoospermia compared to values <6.2 (°). Similarly, a phase angle between 6.2 and 7 (°) (OR: 0.57; p< 0.01 and OR: 0.58; p= 0.01) and PA > 7 (°) (OR: 0.12; p= 0.03 and OR: 0.21; p< 0.01) were associated with a reduced risk of lower sperm concentration and lower total sperm count, respectively, compared to a phase angle < 6.2 (°).

**Conclusion:**

Our study suggests a negative association between phase angle and detrimental sperm parameters in male idiopathic infertility.

## 1 Introduction

In 2020, obesity was a global condition affecting 38% of adults aged 18 years and older (1). The expected global prevalence is projected to increase from 2.6 billion in 2020 to 4 billion by 2035 (1). Furthermore, predictions indicate that the prevalence of overweight in male and female adolescents will rise from 9% in 2020 to 19% in 2035, underscoring the importance of the issue of overweight (1). Similarly, infertility, defined as the inability to achieve pregnancy after 12 months or more of regular unprotected sexual intercourse, has been acknowledged as a significantly widespread global condition, affecting 1 in 6 people at some point in their lives (2). According to the data, up to 50% of infertility in couples can be attributed to male factors (3). Obesity has been demonstrated to be a relevant risk factor for male infertility through various mechanisms including hypogonadotropic-hyperestrogenic hypogonadism, increased testicular inflammation events, resulting from augmented adipokines, raised testicular temperature, sexual dysfunctions and epigenetic induced alteration (4). Altered environment and the subsequent increase in reactive oxygen species (ROS) lead to elevated DNA damage through direct or indirect interaction with the DNA strand (5, 6). For this reason, several studies extensively tested sperm DNA fragmentation (SDF) as a marker of male infertility such as Chavarro and Kort found a statistically significant correlation between augmented and SDF percentage (7) (8),. Their findings were subsequently confirmed by Fariello et al., who observed a higher percentage of damaged DNA in obese men compared to normal-weight and overweight patients (9). Conversely, a recent meta-analysis published in 2020, involving a total cohort of 8255 patients, concluded that the data were insufficient to demonstrate a positive association between overweight and SDF values (10).

A study involving a large group of participants found that male partner obesity increases the risk of infertility for the couple (11, 12). Moreover, when both partners are obese, the risk of infertility is further elevated (13). Despite documented evidence, the relationship between obesity and seminal parameters remains a subject of ongoing debate.

Several studies reported a negative association between overweight and quality of conventional seminal parameters. Belloc et al. found that overweight negatively affects sperm count, sperm concentration, total sperm volume and sperm motility (14). Conversely, according to Aggerholm et al., obesity only affects semen volume but has no effect on other seminal parameters (15–17). To address this endless debate, Guo conducted a meta-analysis involving 26,814 participants and observed that obese patients had no alteration in sperm motility but experienced statistically significant decreases in total sperm count, sperm concentration, and semen volume (18). Since sedentary lifestyle and consequent augmented BMI seems to contribute to male infertility, it is easy to suggest that physical activity has a potentially significant impact on seminal parameters. Indeed, case-control studies have indicated that individuals in the physically active group exhibit enhanced semen parameters, including semen volume, viability, progressive motility, total motility, and morphology, when compared to the sedentary group (19). However, it has been widely reported that, while physical activity is generally associated with improved seminal parameters, excessively intense and prolonged training may have a negative impact on male fertility. Overtraining and intense physical stress may result in hormonal imbalances, elevated testicular temperature, and subsequent oxidative stress, potentially diminishing the quality of semen parameters (20).

Hakonsen reported that obesity-related oligozoospermia can be improved with weight loss, along with enhancements in reproductive hormonal profile and SDF percentage (21, 22) while Andersen observed that increased sperm concentration and total sperm count persist if weight loss is maintained (23).

While BMI is a convenient and easily calculable metric, its indirect estimation of body composition may be prone to inaccuracies. For instance, it can overestimate fat percentage in persons with higher lean muscle mass, such as athletes, and underestimate adiposity in individuals with lower muscular mass (24). To achieve a more accurate evaluation of body composition, various alternative technologies have been developed, with one of the most promising being bioelectric impedance analysis (BIA).

BIA is a validated alternative tool that administers electrical energy through surface electrodes and records tissue responses by measuring parameters such as resistance and reactance (25).The working principle underlying BIA is Ohm’s law, which establishes that the voltage across a conductor is directly related to the resistance to current flow (26). When an electrical current is introduced to biological tissue, its components facilitate the passage of the charge. The predominant charge carriers include mobile ions in water, while the dipolar components, categorized into positive and negative charges, consist of proteins and the lipidic cell membrane. Substantially, the measurement of those electric features gives information about tissues property and composition This permits the prediction of total body water (TBW), fat mass (FM), lean body mass (LBM), and the percentage of body fat (%BF), providing an accurate estimation of adiposity (27, 28). Phase angle (PA) is a function of the relationship between resistance and reactance representing a measure of extra- and intracellular water content; since electricity flows more easily through hydrated tissue, such as muscle, PA seems the most trustworthy predictor of body composition (25, 29). Phase angle in the context of bioimpedance refers to the phase shift between the voltage and current in an electrical circuit that passes through biological tissues. BIA is a method used to measure the impedance of biological tissues to alternating electrical currents. This impedance includes both resistive (real) and capacitive/reactive (imaginary) components. Moreover, PA represents a reliable marker of membrane integrity and cell mass (30). Essentially, BIA relies on models that predict TBW as a linear function of the resistance index, considering factors such as weight, age, and gender (31). However, literature data are missing regarding the potential association between PA and sperm parameters in patients with infertility. For these reasons, our study aims to assess the existence of an association between PA and seminal parameters, including total sperm count, sperm concentration, total motility, morphology, and SDF percentage in patients with idiopathic male infertility.

## 2 Materials and methods

A total of 520 consecutive male patients, seeking assistance at the Unit of Reproductive Medicine in the clinic ‘Centro HERA’ for primary couple infertility, participated in this prospective study (from January 2023 to June 2023. Our study included patients aged 18 years or older who were affected by idiopathic infertility. We collected information on the patients’ age and conducted a physical examination, documenting measurements of height, weight, and BMI. Each patient performed sperm analysis, evaluation of SDF and BIA before starting any other treatment. Patients with varicocele, male accessory gland infection, genetic alterations, and hormonal diseases were excluded from the study. The current study protocol obtained approval from the Institutional Review Board at Centro HERA - UMR (Approval No. 1/2023). All subjects provided informed consent upon enrollment in the study.

### 2.1 Sperm analysis

Semen samples were collected through masturbation into a sterile container following 2–7 days of sexual abstinence. Analysis was conducted immediately after liquefaction. Each sample was assessed for seminal volume, sperm count, progressive motility, and morphology, in accordance with the 2021 WHO guidelines (3).

### 2.2 Sperm DNA fragmentation

Sperm samples underwent terminal deoxynucleotidyl transferase-mediated dUTP-digoxigenin nick end-labelling (TUNEL) staining using a commercially available kit (Dead End Fluorimetric TUNEL System; Promega, USA) following the manufacturer’s instructions. Briefly, sperm were fixed in 4% paraformaldehyde at 4°C and permeabilized with 0.2% Triton X-100 (Promega) in PBS (Nutricell). After permeabilization, the samples were incubated in 100 ml drops with a reagent mix containing terminal deoxynucleotide transferase enzyme solution and 90% staining solution (dUTP fluorescein conjugate) for 1 h at 37°C in a dark humid chamber. Subsequently, the sperm were stained with Vectashield (Vector Laboratories Inc., Burlingame, CA, USA), plus 4’,6-diamidino-2-phenylindole (DAPI) and mounted on slides for evaluation using fluorescence microscopy (Olympus BX51). The TUNEL assay results were reported as the percentage of sperm DNA fragmentation, indicating the proportion of cells with DNA damage (32).

### 2.3 Bioelectric impedance analysis

Participant body composition was assessed during a single-day visit (<1 hour). Individuals were assessed on a direct segmental octopolar multi-frequency device (InBody, Model 770, Cerritos, California, USA), standing with feet apart and elbows extended to avoid body contact for approximately 1 min. The bare feet made positive contact with the base electrodes at the heels and forefeet and subjects grasped two handle electrodes for direct contact with two more electrodes for each hand at thumbs and forefingers. The segmental analysis was computed with proprietary algorithms. Data obtained from the InBody 720 device were processed using the Lookin Body 3.0 program. By data analysis, biometric information for each patient were collected, including:

- Fat mass (FM).
- Lean mass (LM).
- Muscular mass (MM).
- Percentage of body mass (%BF).
- Waist to hip ratio (WHR).
- Abdominal circumference (AR).
- PA (33).

Subjects reported to the laboratory for a single testing session after a minimum of 08 hours of fasting from food, caloric beverages, caffeine, alcohol, and tobacco. Additionally, subjects refrained from strenuous exercise for a minimum of twelve hours before testing. Height (cm) and weight (Kg) were measured upon arrival at the laboratory using a calibrated scale. For all measurements, subjects were instructed to be free from metal (e.g., zippers, jewelry, hard plastic) to avoid interference with data collection accuracy. Multi-frequency bioelectrical impedance analysis (MF-BIA) using the InBody 770 device (Biospace Co.) estimated total body composition, including fat percentage, FM and LM. Subjects stood barefoot on the device’s scale for 5 minutes, with the soles of their feet positioned on four corresponding electrodes and holding the handles in both hands to contact corresponding electrodes on the thumbs and palms. Height, sex, and age were entered into the MF-BIA software, and the device collected weight. Subjects remained still for the duration of the assessment.

### 2.4 Statistical analysis

All statistical analyses were conducted using Stata (Stata Statistical Software: College Station, TX: Stata Corp LP). For all statistical comparisons, results were considered significant when p < 0.05. Normally distributed continuous variables were presented as median (interquartile range, IQR), and differences between groups were tested by Student’s independent t-test or Mann–Whitney U-test, depending on their normal or non-normal distribution (normality of variables’ distribution was tested by Kolmogorov–Smirnov test).

Age-adjusted linear regression models were performed to verify factors correlated with abnormal sperm parameters, expressed as beta-coefficient. The beta-coefficient represents the magnitude of the variation in the independent variable for each increase in the dependent value.

Multivariable logistic regression models were constructed to identify predictive factors of:

- Oligozoospermia, defined as < 39 million or < 15 million/ml of spermatozoa.
- Asthenospermia, defined as motility lower than 32%.
- Teratozoospermia, defined as normal morphology lower than 4%.
- Oligoasthenoteratozoospermia (OAT), defined as the coexistence of these abnormalities.

PA (reference value from 1 to 10) has been categorized into three sub-groups according to the resulting tertiles:

1) PA <6.2;
2) PA between 6.2 and 7;
3) PA >7.

A cut off of 20% has been considered for SDF according to Agarwal (34). Area under the curve (AUC) has been performed to verify accuracy of phase angle in diagnosing OAT.

## 3 Results

The median age was 40 years old (interquartile range [IQR]: 37.0-45.0), and the median BMI was 26.3 kg/m2 (IQR: 24.2 – 29.3). Additionally, patients’ biometric parameters were collected: median FM was 20.15 kg (IQR: 13.1 – 24.8), median percentage of fat mass was 22.95% (IQR: 17.7-27.9), median LM was 62.3 kg (IQR: 57.3 – 67.5), median MM was 36.25 kg (IQR: 33.1 – 38.8), median AC was 96.35 cm (IQR: 86.3 – 103.6), median WHR was 0.945 (IQR: 0.89- 0.98), and median PA was 6.2°(IQR: 5.8 – 6.5). Baseline characteristics of the entire cohort are listed in **Table 1**.

**Table 1:** Baseline characteristics of the study cohort (*n=520*).

Age (years old), median (IQR)	40 (37 – 45)
Weight (Kg), median (IQR)	83.1 (74.4 – 92.1)
Height (cm), median (IQR)	177 (173 – 180)
BMI (Kg/m^2^), median (IQR)	26.3 (24.2 – 29.3)
FM (Kg), median (IQR)	20.15 (13.1 – 24.8)
LM (Kg), median (IQR)	62.3 (57.3 – 67.5)
MM (Kg), median (IQR)	36.25 (33.1 – 38.8)
%BF (%), median (IQR)	22.95 (17.7 – 27.9)
WL (cm), median (IQR)	88.85 (57.9 – 109.2)
AC (cm), median (IQR)	96.35 (86.3 – 103.6)
WHR, median (IQR)	0.945 (0.89 - 0.98)
PA (°), median (IQR)	6.2 (5.8 – 6.5)
Total sperm count (Mil), median (IQR)	67.16 (30.8 – 130.63)
Sperm concentration (Mil/ml), median (IQR)	25 (9.2 – 48.0)
Total motility (%), median (IQR)	15.5 (5 – 26)
Morphology (%), median (IQR)	6 (4 – 8)
SDF (%), median (IWR)	22 (16 – 29)

BMI, Body mass index; FM, Fat mass; LM, Lean mass; MM, Muscular mass; %BF, Percentage of Body Fat; WL, Waistline; AC, Abdominal circumference; WHR, Waist to hip ratio; PA, Phase angle; SDF, Sperm DNA fragmentation.

Classification of the analyzed cohort according to BMI in presented in **Supplementary Table 1**.

All semen analyses were conducted on sperm samples obtained after a median day of ejaculatory abstinence of 4 days (IQR: 3- 4). The semen analysis reported a median SDF of 22.0% (IQR: 16.0-29.0), median sperm concentration of 25.0 million/ml (IQR: 9.2-48.0), median total sperm count of 67.16 million (IQR: 30.8-130.63), median progressive motility of 15.5% (IQR: 5.0-26.0), and median morphology of 6.0% (IQR: 4.0-8.0). A total of 116 patients (22.3%) suffered from OAT. The prevalence of the remaining semen alterations among the cohort is listed in **Supplementary Table 2**.

Patients with PA ≤ 6.2 had higher median age (42 vs 40; p<0.01), lower median BMI (25.7 vs 26.3; p< 0.01), lower median lean mass (60.2 vs 62.3; p<0.01), lower median muscular mass (34.0 vs 36.2; p<0.01), lower median abdominal circumference (92.8 vs 96.35; p<0.01). Other biometric data did not show significant differences between the two groups. Moreover, patients with PA ≤ 6.2 had a significantly lower total sperm count (60.6 vs 67.16; p<0.05). The other sperm parameters were not significantly influenced by PA variation (**Table 2**).

**Table 2:** Anthropometric characteristics and sperm parameters according to the phase angle.

	PA > 6.2	PA ≤ 6.2	P-value
Age (years), median (IQR)	38 (35.0 – 42.0)	42 (38.0 – 46.0)	<0.01
Weight (Kg), median (IQR)	86.9 (77.6 – 94.6)	79.2 (71.7 - 90)	<0.01
Height (cm), median (IQR)	177 (173 -180)	178 (173 – 180)	0.48
BMI (Kg/m^2^), median (IQR)	27.7 (25.6 – 29.7)	25.7 (22.4 – 28.4)	<0.01
FM (Kg), median (IQR)	20.4 (14.9 – 25.3)	18 (11.7 – 24.5)	0.08
LM (Kg), median (IQR)	65.1 (60.4 – 70.7)	60.2 (54.7 – 65.9)	<0.01
MM (Kg), median (IQR)	37.6 (35.3 – 40.6)	34.0 (31.3 – 37.3)	<0.01
%BF (%), median (IQR)	23.0 (18.7 – 27.5)	22.8 (17.1 – 30.2)	0.74
WL (cm), median (IQR)	91.2 (63.5 – 110-0)	82.7 (53.6 – 109.2)	0.50
AC (cm), median (IQR)	99.2 (90.1 – 106.0)	92.8 (84.5 – 102.1)	<0.01
WHR, median (IQR)	0.95 (0.9 - 0.99)	0.94 (0.88 - 0.98)	0.10
Sperm volume (ml), median (IQR)	7.42 (3.54 – 14.52)	6.06 (2.76 – 12.62)	0.04
Sperm concentration (million/ml), median (IQR)	28.05 (12.6 – 48.0)	21.45 (8.25 – 49.5)	0.20
Progressive motility (%), median (IQR)	15.0 (6.0 – 27.0)	16 (5.0 – 26.0)	0.81
Morphology (%), median (IQR)	5.0 (4.0 – 8.0)	6.0 (4.0 – 8.0)	0.37
SDF (%), median (IQR)	24.0 (14.0 – 29.0)	22 (17.0 – 29.0)	0.22

PA, Phase angle; BMI, Body mass index; FM, Fat mass; LM, Lean mass; MM, Muscular mass; %BF, Percentage of Body Fat; WL, Waistline; AC, Abdominal circumference; WHR, Waist to hip ratio; SDF, Sperm DNA fragmentation.

**Figure 1:**
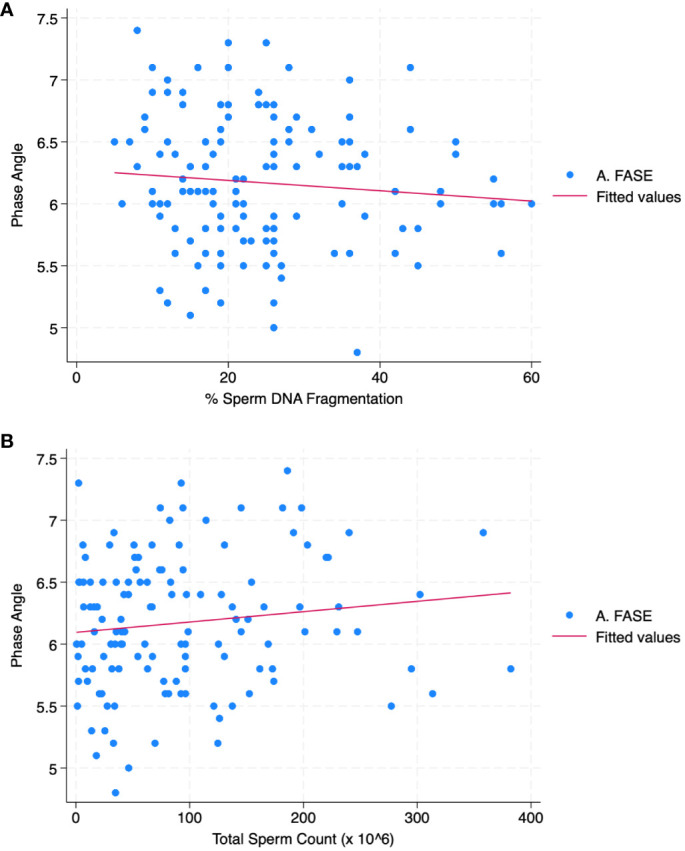
Scatter plot graph between phase angle and SDF **(A)** and TSC **(B)**.

**Table 3** reports the correlation analysis between all parameters. PA was correlated with SDF (r = - 0.09; p< 0.05) and total sperm count (r = 0.12; p< 0.01). **Figures 1A, B** shows the scatter plot of the association between phase angle and SDF (**Figure 1A**) and TSC (**Figure 1B**).

**Table 3:** Correlation analysis between parameters.

	Age	BMI	FM	LM	MM	WL	AC	WHR	Wtot	Wic	Wec	PA	SDF	Sperm concen-tration	Total sperm count	Motility	Morphology
Age	1.00	-0.01	0.09*	0.13**	-0.09*	0.10*	0.05	0.11*	-0.13**	-0.14**	-0.09	-0.33**	0.25**	0.08	-0.07	-0.14**	0.04
BMI	-0.01	1.00	0.91**	0.58**	-0.09*	0.9**	0.9**	0.79**	0.55**	0.52**	0.53**	0.33**	-0.02	-0.03	-0.01	-0.03	-0.01
FM	0.09*	0.91**	1.00	0.44**	0.42**	0.99**	0.96**	0.91**	0.42**	0.43**	0.42**	0.11**	0.02	0.13	0.01	-0.02	0.01
LM	0.13**	0.58**	0.44**	1.00	0.91**	0.44**	0.56**	0.39**	0.97**	0.89**	0.96**	0.27**	0.05	-0.15**	-0.05	-0.01	-0.07
MM	-0.09*	0.55**	0.42**	0.91**	1.00	0.42**	0.55**	0.38**	0.93**	0.89**	0.92**	0.35**	0.06	-0.14**	-0.05	0.01	-0.02
WL	0.10*	0.9**	0.99**	0.44**	0.42**	1.00	0.96**	0.92**	0.42**	0.43**	0.42**	0.06	-0.01	0.01	0.01	-0.01	-0.01
AC	0.05	0.9**	0.96**	0.56**	0.55**	0.96**	1.00	0.95**	0.56**	0.57**	0.54**	0.22**	-0.02	-0.01	0.01	-0.01	0.01
WHR	0.11*	0.79**	0.91**	0.39**	0.38**	0.92**	0.95**	1.00	0.39	0.42**	0.37**	0.12**	-0.03	0.04	0.03	0.02	0.02
Wtot	-0.13**	0.55**	0.42**	0.97**	0.93**	0.42**	0.56**	0.39	1.00	0.94**	0.98**	0.3**	0.06	-0.14**	-0.04	0.01	0.04
Wic	-0.14**	0.52**	0.43**	0.89**	0.89**	0.43**	0.57**	0.42**	0.94**	1.00	0.92**	0.35**	0.04	-0.09*	0.01	0.07	0.02
Wec	-0.09	0.53**	0.42**	0.96**	0.92**	0.42**	0.54**	0.37**	0.98**	0.92**	1.00	0.18**	0.08*	-0.16**	-0.06	0.01	-0.05
PA	-0.33**	0.33**	0.11**	0.27**	0.35**	0.06	0.22**	0.12**	0.3**	0.35**	0.18**	1.00	-0.09*	0.04	0.12**	0.02	0.1*
SDF	0.25**	-0.02	0.02	0.05	0.06	-0.01	-0.02	-0.03	0.06	0.04	0.08*	-0.09*	1.00	-0.04	-0.1*	-0.07	-0.08
Sperm Concentration	0.08	-0.03	0.13	-0.15**	-0.14**	0.01	-0.01	0.04	-0.14**	-0.09*	-0.16**	0.04	-0.04	1.00	0.76**	0.47**	0.63**
Total sperm count	-0.07	-0.01	0.01	-0.05	-0.05	0.01	0.01	0.03	-0.04	0.01	-0.06	0.12**	-0.1*	0.76**	1.00	0.54**	0.58**
Motility	-0.14**	-0.03	-0.02	-0.01	0.01	-0.01	-0.01	0.02	0.01	0.07	0.01	0.02	-0.07	0.47**	0.54**	1.00	0.56**
Morphology	0.04	-0.01	0.01	-0.07	-0.02	-0.01	0.01	0.02	0.04	0.02	-0.05	0.1*	-0.08	0.63**	0.58**	0.56**	1.00

BMI, Body Mass Index; FM, Fat mass; LM, Lean mass; MM, Muscular mass; WL, Waistline; AC, Abdominal circumference; WHR, Waist to hip ratio; Wtot, Total water; Wic, Intracellular water; Wec, Extracellular water; PA, Phase angle; SDF, Sperm DNA fragmentation.

*p-value <0.05.

**p value <0.01.

The age-adjusted linear regression analysis demonstrated that PA was positively associated with sperm count (r=0.01; p=0.01) and morphology (r=0.02; p<0.01) but not with SDF (p=0.81), sperm concentration (p=0.06) and total motility (p=0.58).

In the logistic regression analysis adjusted for age and total intracorporeal water, PA (continuous) (OR [odds ratio]: 0.4; 95%CI 0.27-0.59; p<0.01) was significantly associated with oligozoospermia but not with the SDF (OR: 0.98; p=0.07) and with sperm morphology (OR: 0.65; p=0.05).

Logistic regression analysis of PA sub-groups, adjusted for age, total intracorporeal water, and SDF showed that a PA between 6.2 and 7 (°)(OR: 0.63; 95%CI 0.42-0.94; p=0.02) and PA >7 (°)(OR: 0.12; 95%CI 0.04-0.37; p<0.01) were associated with reduced risk of oligozoospermia compared to PA <6.2 (°). Similarly, PA between 6.2 and 7 (°)(OR: 0.57; 95% CI 0.37- 0.86; p< 0.01 and OR:0.58 95% CI 0.38- 0.88; p= 0.01) and PA > 7 (°)(OR: 0.12; 95% CI 0.0.4- 0.36; p= 0.03 and OR: 0.21; 95% CI 0.07- 0.63; p< 0.01) were associated with reduced risk of lower sperm concentration and of lower total sperm count respectively, compared to PA < 6.2 (°).

PA sub-groups logistic regression analysis adjusted for age and total intracorporeal water did not show any relation with sperm morphology (p=0.98 and p=0.09).

The AUC for phase in angle in diagnosing OAT was 0.61.

## 4 Discussion

Herein our data suggests that patients with lower PA (°) (≤ 6.2) had detrimental sperm parameters in particular lower sperm concentration and total sperm count. Our study is the first investigating the potential association between PA and low sperm quality in patients with idiopathic male infertility.

Although the exact mechanism by which PA influences sperm parameters remains unclear, several hypotheses have been proposed. First, PA is employed to assess nutritional status and body composition based on the electrical properties of different tissues (35). Indeed, since electricity flows more easily through hydrated tissue, such as muscle, it is foreseeable that suitable PA values are predictors of better body composition (29). On the other hand, some studies claim that BIA has a limited accuracy in predicting body composition (36). BIA appears to be significantly influenced by environmental factors, ethnicity, and medical conditions. Therefore, the development of an appropriate calibration equation is necessary for different groups of participants (37).

The relationship between phase angle from BIA and sperm DNA fragmentation has not been extensively studied or validated. While BIA has been investigated in various clinical contexts, it’s utility in predicting sperm DNA fragmentation remains largely unexplored. Sperm quality and DNA integrity are influenced by multiple factors beyond cellular health, including oxidative stress, exposure to toxins, lifestyle factors, and genetic factors. While BIA may provide some insights into overall health status, it may not capture all the determinants of sperm quality and DNA fragmentation.

However, since PA is a measure of extra and intracellular water content, it serves as a direct indicator of cell membrane integrity; in fact, the smaller the PA, the weaker the cell structure, and the higher the probability of cell death (38). Various studies have reported an association between PA and biochemical markers involved in monitoring chronic diseases as well as cancer prognosis (39, 40). Given that chronic diseases promote inflammation and oxidative stress, which may be responsible for cell damage, PA can serve as an early predictor of inflammation (41). Among chronic disorders, obesity is reported to be a noteworthy promoter of inflammatory status, characterized by an increase in tumor necrosis factor-α (TNF-α), interleukin 6 (IL6), and interleukin 10 (IL10) production (42). Building upon this previous statement, patients with a higher BMI are more likely to exhibit cell membrane damage, contributing to cell fluid imbalance and lower PA values (43).

Based on these premises and considering that chronic oxidative stress is known to affect sperm quality by damaging sperm DNA, PA appears to be a promising predictor of semen quality (44).

To establish phase angle as a strong predictor of male fertility, a multifaceted approach is necessary. Firstly, extensive research is paramount. Investigating the relationship between phase angle and male fertility demands thorough exploration. This entails collecting data from diverse populations to ensure the reliability and universality of findings. Collaboration with experts across various disciplines such as nutrition, physiology, endocrinology, and reproductive medicine is essential. Their insights can illuminate the underlying mechanisms linking phase angle with male fertility, enriching our understanding. Developing diagnostic tools or algorithms that integrate phase angle measurements with other pertinent biomarkers is pivotal. These tools should accurately assess male fertility status, enhancing diagnostic precision. Validation and standardization efforts are indispensable. Validating phase angle’s predictive capacity across diverse populations and settings ensures its reliability. Standardizing measurement protocols and interpretation criteria promotes consistency and reproducibility of results. By pursuing these comprehensive steps, phase angle can emerge as a robust predictor of male fertility, facilitating more accurate diagnosis and management of male infertility issues.

Limitations of the study are important to be defined. Infertility often results from a combination of factors, including hormonal imbalances affecting ovulation or sperm production, structural issues impacting the reproductive organs, and systemic health conditions affecting fertility. Phase angle may not capture the multifaceted aspects contributing to infertility. The relationship between phase angle and infertility lacks extensive study and validation. While phase angle has been explored in various clinical contexts such as nutritional status, muscle health, and disease prognosis, its utility in predicting infertility remains largely unexplored. BIA measurements, including phase angle, can be influenced by external factors like hydration status, body temperature, skin integrity, and electrode placement. Fluctuations in these factors can affect the accuracy and reliability of BIA measurements, potentially complicating the interpretation of phase angle in predicting infertility. Individuals exhibit significant variability in phase angle values based on factors such as age, sex, body composition, and overall health status. Additionally, reference ranges for phase angle may vary across different populations and measurement techniques, making it challenging to establish universal thresholds for predicting infertility based on phase angle alone.

In summary, our data suggest an association between PA and semen parameters; however, further research is needed to fully understand the underlying mechanisms. Furthermore, a limitation of our study was the lack of data regarding the lifestyle habits and comorbidities of our patients, which may be of great relevance when evaluating fertility potential and alterations in semen parameters.

## 5 Conclusion

In conclusion, further research is needed to fully understand the association between semen parameters and PA. Nevertheless, this preliminary study suggests that the phase angle assessed at BIA may be associated with poor sperm quality in males affected by idiopathic infertility. Our preliminary data may support further studies that can reveal the impact of phase angle with other aspects of couple infertility in the context of assisted reproductive technology. Furthermore, these results highlight the detrimental relationship between abnormal body composition and sperm quality. Clinicians may consider these results when developing strategies to increase the phase angle and improve semen quality.

## Data availability statement

The raw data supporting the conclusions of this article will be made available by the authors, without undue reservation.

## Ethics statement

The studies involving humans were approved by Institutional review board of Centro HERA - UMR “Unità di Medicina della Riproduzione”. The studies were conducted in accordance with the local legislation and institutional requirements. The participants provided their written informed consent to participate in this study.

## Author contributions

GR: Conceptualization, Data curation, Formal analysis, Investigation, Methodology, Project administration, Supervision, Validation, Visualization, Writing – review & editing. AL: Conceptualization, Data curation, Investigation, Methodology, Validation, Visualization, Writing – review & editing. FG: Conceptualization, Data curation, Investigation, Methodology, Validation, Visualization, Writing – review & editing. DL: Investigation, Validation, Visualization, Writing – review & editing. MA: Formal analysis, Investigation, Validation, Visualization, Writing – original draft, Writing – review & editing. AA: Writing – review & editing, Formal analysis. SCi: Investigation, Validation, Visualization, Writing – review & editing. AG: Investigation, Validation, Visualization, Writing – review & editing. SCh: Investigation, Validation, Visualization, Writing – review & editing.
