# Supplementary material for: Phase angle at bioelectric impedance analysis is associated with detrimental sperm quality in idiopathic male infertility: a preliminary clinical study
Source: Front Endocrinol (Lausanne). 2024 Apr 24;15:1354733. doi: 10.3389/fendo.2024.1354733 (PMC11076772; doi:10.3389/fendo.2024.1354733)
Supplement: Supplementary file 1 [file Table_1.docx]

Supplementary tables

# Supplementary tables

| **Supplemental Table 1. Proportions of patients according to their BMI** | |
| --- | --- |
| Healthy weight (BMI 18.5-24.9 Kg/m^2^), n (%) | 164 (31.54%) |
| Underweight (BMI < 18 Kg/m^2^), n (%) | 8 (1.54%) |
| Overweight (BMI 25-29.9 Kg/m^2^), n (%) | 252(48.46%) |
| Obesity class 1 (BMI 30-34.5 Kg/m^2^), n (%) | 76 (14.62%) |
| Obesity class 2 (BMI > 35 Kg/m^2^), n (%) | 20 (3.85%) |
| BMI = body mass index | |

| **Supplemental Table 2. Proportions of patients according to their according to semen parameters alterations according to WHO 2021** | |
| --- | --- |
| Oligozoospermia, n (%) | 220 (42.31%) |
| Teratozoospermia, n (%) | 124 (23.85%) |
| Astenozoospermia, n (%) | 456 (87.69%) |
| Oligoastenoteratozoospermia, n (%) | 116 (22.31%) |
| WHO = World Health Organization | |
